# Case of Colica Pictonum

**Published:** 1853-03

**Authors:** C. A. Hathwell

**Affiliations:** Virginia, Ill.


ART. II —*Case of Colica Pictonum, resulting from the use of Lead Plaster.* By C. A. Hathwell, M.D., of Virginia, Ill.

The latter part of March, 1852, I was called upon by H. E· W., and requested to examine his "sore leg", stating that he had used "everything and nothing would cure it up". he removed the bandages and exhibited an extensive ulceration of the Tibia in a foul, dirty, and irritable condition.

He informed me that he had been afflicted with it a long time, and wished I would "heal it right up." After examination I ordered him to wash it perfectly clean with warm water and castile soap, apply a solution of nitrate of silver, then poultice it, to keep the leg inclined and at rest till I saw him again; all this he made objections to, upon the grounds of wishing to "go to no great expense, as it had been bad so long;" he further remarked, that as he had to break up ground and go to ploughing, he only wanted "summut" to keep the dust and dirt away from it. Seeing him determined to prescribe for himself, I told him to wash it, gave him a quantity of emp. plumbi, with directions, and he left the office.

I saw no more of him till July, when I was summoned to attend him. He was in bed on my arrival; I found him with much gastric derangement, obstinate constipation, nausea, languor, constant thirst, great anxiety of the countenance, pulse quick, tenderness of the abdomen, pain about the umbilicus, &c. From the symptoms, I judged it a case of Lead, Painter's, or Devonshire colic, as described by authors. I pursued the following treatment: ℞ Pulv. opii gr. viii Hyd. Mur. Mit. ʒij. M. Div. in Pulv. No. iv, one every three hours, or until subsidence of abdominal pains, to apply flannel clothes wrung out of hot alcohol, and to be followed up with a full dose of oil and turpentine. Saw him again next day, was somewhat relieved, but had no operation on the bowels, ordered an injection of infus. Sennæ; this produced the desired effect, and afforded my patient much relief. During my visit I enquired of him how his sore leg progressed? he answered that it was not much, if any, better than when I had seen it in March. I examined it, and found it in a highly irritable and inflamed state, and still covered with the lead plaster as I had ordered four months previously. I directed him to wash it and apply poultices; in a short time he regained his general health, and I ceased attendance on him.

I heard nothing of the patient till November, when that nocturnal "hollo!" so unpleasant to the ears of a physician, one night admonished me to arise. Having but just recoverd myself from a severe attack, I very reluctantly abandoned my downy pillow and obeyed the orders of the messenger, who again summoned me to H. E. W. On questioning him, I observed that he was suffering under the same, or nearly the same, symptoms as previously. The pain in the abdomen, the costiveness, the bent position of my patient &c., enabled me to form my diagnosis and I treated the case accordingly. Being the first case of Colica Pictonum I had seen in Illinois during a residence of ten years, I was somewhat curious in my inquiries respecting its origin, but received no satisfaction from the patient himself. I then examined his ulcerated leg, and found him truly faithful as regarded the application of the diachylon; there it was, and had been ever since I had formerly ceased to visit him. It now occurred to my mind, that the protracted use of the lead plaster might possibly be the original source of his complaint, so I immediately ordered its discontinuance.

To be brief, the case readily yielded to appropriate remedies, and he recovered in due time; he peremptorily refused to have anything done for his leg, observing that he had "tried all the Doctors and none of 'em could'nt do him no good." He has had no return of the disease up to the present time (February) and has relinquished the diachylon. In my own mind, I am fully satisfied his complaint was produced by the long continued application of the Emp. Plumbi, but how? by absorption? or the result of decomposition?

